# Absolute B cell counts in blood predict long-term response in follicular lymphoma patients treated with rituximab without chemotherapy

**DOI:** 10.1007/s00277-020-04208-x

**Published:** 2020-08-17

**Authors:** Henna-Riikka Junlén, Sandra Lockmer, Eva Kimby, Björn Engelbrekt Wahlin

**Affiliations:** 1 Unit of Hematology, Department of Medicine, Karolinska Institutet, Huddinge, Stockholm Sweden; 2 Medicinsk enhet Hematologi, Tema Cancer, Karolinska University Hospital, Stockholm, Sweden

**Keywords:** Follicular lymphoma, Rituximab, B cell, Monocyte, Lymphocyte

## Abstract

Rituximab monotherapy is widely used for follicular lymphoma. However, there are no established predictors for response or response duration. We analyzed the long-term prognostic relevance of pre-treatment absolute blood counts of lymphocytes with subsets and monocytes in 265 follicular lymphoma patients, uniformly treated with rituximab without chemotherapy, in two Nordic Lymphoma Group trials. There were 265 previously untreated, stage II–IV follicular lymphoma patients with a median follow-up of over 10 years. Absolute B cell counts ≥ median (0.09 × 10^9^/L) were an independent predictor for shorter time to next treatment or death (multivariable analysis *P* = 0.010). In univariate analysis, absolute monocyte counts ≥ median (0.5 × 10^9^/L) did not correlate with time to next treatment or death, but with inferior overall survival (*P* = 0.034). Absolute T cell or T cell subset counts were not predictive for outcome. High absolute B cell counts, possibly reflecting circulating lymphoma cells, have an unfavorable impact on time to next treatment or death in patients treated with rituximab without chemotherapy.

## Introduction

Follicular lymphoma (FL) is the most common indolent non-Hodgkin lymphoma, and it is characterized by a highly variable clinical course [1]. FL consists of clonal centrocytes and centroblasts on whose numbers the World Health Organization (WHO) allocates FL to grades 1, 2, and 3A [1]. Standard first-line treatment is rituximab alone or in combination with chemotherapy.

The Follicular Lymphoma International Prognostic Index (FLIPI) is the most common system for risk stratification of FL, also in the rituximab era [2, 3]. However, the FLIPI cannot guide the choice of therapy and better prognostic and predictive tools are needed. Non-malignant immune cells in the FL tumor microenvironment, supporting growth and survival of tumor cells and suppressing the antitumor immune response, also have prognostic relevance [4]. The host immune system is important for the clinical effect of therapy with the anti-CD20 antibody rituximab, as the effect of rituximab is dependent on T cells, NK cells, and monocytes/macrophages [5, 6]. The absolute monocyte count in blood has been proposed as an indicator for the level of tumor-induced immunosuppression, and for use as a possible prognostic marker in FL and other lymphomas [7–11]. The absolute lymphocyte count has also been suggested to have prognostic value in FL [12–14] and other lymphomas [15–18].

In two Nordic Lymphoma Group (NLG) chemotherapy-free trials using rituximab, we have previously reported that higher relative T cell levels in blood and in diagnostic lymph nodes predict better outcome including time to next treatment or death (TNT) [5]. Also higher WHO FL grades (2–3A) seemed favorable in comparison to WHO FL grade 1 [19]. Here, we have assessed the prognostic value of absolute lymphocyte subset and monocyte counts.

## Methods

The study cohort consists of 265 previously untreated FL patients with absolute lymphocyte count and absolute monocyte count available from routine blood cell counts taken prior to the start of first-line therapy. The patients were part of the total study population of 321 patients with indolent lymphoma (269 with FL, 27 with marginal zone lymphoma, 3 with lymphocytic lymphoma, and 22 with low-grade lymphoma not otherwise specified) participating in two consecutive randomized NLG trials M39035 (phase II, inclusion 1998–1999) [20] and ML16865 (phase III, inclusion 2002–2008) [21] with published data on long-term follow-up [22]. In these trials, patients received two cycles consisting of four doses of rituximab 375 mg/m^2^ and they were 1:1 randomized to the addition of interferon-α2a (IFN). Diagnostic tumor biopsies were centrally reviewed according to the WHO Classification (FL grades 1–3A) [1], and bone marrow involvement was evaluated by the local pathologist. All patients were ≥ 18 years old and had advanced (stage II–IV) disease, WHO performance status 0–2, and indication for therapy due to symptomatic or progressive disease.

In the clinical trial protocols, pre-treatment flow-cytometry analysis of blood and bone marrow was recommended but not obligatory. Flow cytometry of blood and bone marrow was performed in 132 and 192, respectively, of the 265 patients, and data on both specimens was available in 114 subjects. The lymphocyte subsets were reported as percentages of the population within the mononuclear gate. B cells were defined as CD19+ cells (or CD20+, in a few cases where the CD19 analysis was missing), T cells as CD3+, helper T cells as CD4+, cytotoxic T cells as CD8+, and NK cells as CD56+. Detailed descriptions of the flow-cytometry analysis and central pathology review have been published earlier [5, 19].

The clinical trials were conducted in accordance with the Declaration of Helsinki and the national laws and regulations concerning clinical trials. Written informed consent was obtained from all study participants prior to the clinical trial enrollment. For the present analysis, we used clinical data from a recently published, ethically approved, follow-up study [22]. The addition of IFN did not affect long-term outcome in the clinical trials [20–22].

### Statistical analysis

TNT was calculated from the date of trial inclusion to the date of next anti-lymphoma therapy or death of any cause. Overall survival (OS) was calculated from the date of trial inclusion to the date of death. Associations with TNT and OS were estimated using the Kaplan-Meier method and the Cox proportional hazards analysis. The assumption of proportionality was checked using Schoenfeld’s residuals. Correlations between variables were analyzed using the Mann-Whitney-Wilcoxon, Spearman, Fisher’s exact test, and logistic regression depending on the nature of the variables. The blood cell counts and the lymphocyte subsets were tested as continuous as well as binary variables (dichotomized by the median) and also divided into tertiles. WHO FL grades were analyzed as a binary variable: WHO FL grade 1 as one group and WHO FL grades 2 and 3A as the second group, due to a low number of patients with WHO FL grade 3A (*N* = 13). Institutional upper normal limits (UNL) were used as cut-off values for lactate dehydrogenase (LDH), where we made an exploratory analysis of LDH as a three-group categorical variable (LDH < 1 × UNL, LDH 1–1.49 × UNL, and LDH ≥ 1.50 × UNL). All *P* values are two-tailed and *P* < 0.05 was considered significant. All statistical calculations were performed using Stata 14.2 (StataCorp, College Station, TX, USA).

## Results

There were 265 FL patients treated in first line with rituximab without chemotherapy in the two trials. The median age was 56 years, 51% were women, and 78% had intermediate or high-risk FLIPI (Table 1). Nineteen (7.2%) of the patients had lymphocytosis (> 4 × 10^9^/L) and 62 (23.4%) lymphopenia (< 1.0 × 10^9^/L) according to the current definitions of lymphocytosis and lymphopenia in adults [23]. Rituximab monotherapy was given to 146 patients and rituximab combined with IFN to 119. The median follow-up time for surviving patients was 10.6 years (range, 0.3–18.8 years). The median TNT was 2.2 years, with 184 patients having received new therapy.

**Table 1:** Clinical characteristics and their relation to outcome

Variable	*N*	%	TNT		OS	
			*P*	HR (95% CI)	*P*	HR (95% CI)
Male sex	129	48.7	0.75		0.34	
Age > 60 years	84	31.7	0.23		0.001	2.35 (1.45–3.80)
Median (range): 56 (23–82) years						
Ann Arbor stage III–IV	237	89.2	0.98		0.17	
Involved nodal areas > 4	155	58.5	0.36		0.09	0.66 (0.41–1.07)
Hemoglobin ≤ 12 g/dL	49	18.5	0.52		0.18	
LDH > UNL	74	27.9	0.0004	1.75 (1.28–2.38)	0.020	1.80 (1.10–2.94)
FLIPI intermediate risk	104	39.3	0.13	1.35 (0.91–2.01)	0.70	
FLIPI high risk	102	38.5	0.021	1.59 (1.07–2.35)	0.27	
Bone marrow involvement	129	51.8	0.46		0.47	
Bulky disease	48	18.1	0.30		0.56	
B symptoms	69	26.0	0.022	1.44 (1.05–1.98)	0.09	1.54 (0.93–2.55)
WHO performance status 1–2	68	25.7	0.08	1.34 (0.97–1.84)	0.0002	2.53 (1.56–4.12)
WHO grade 1	122	46.0	0.009	1.46 (1.10–1.94)	0.046	1.63 (1.01–2.65)
Elevated lymphocytes (> 4 × 10^9^/L)	19	7.2	0.008	1.96 (1.19–3.23)	0.19	

*TNT*, time to next treatment or death; *OS*, overall survival; *HR*, hazard ratio; *CI*, confidence interval; *LDH*, lactate dehydrogenase; *UNL*, upper normal limit; *FLIPI*, Follicular Lymphoma International Prognostic Index; WHO, World Health Organization

**Fig. 1:**
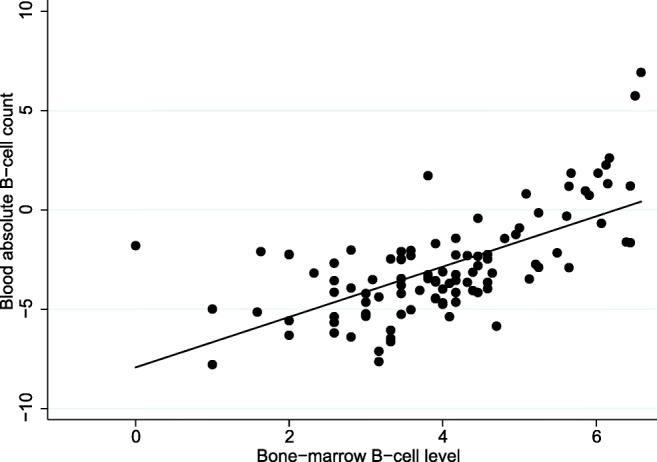
Correlation of B cells in bone marrow and blood. Scatter plot of the correlation between flow cytometry-derived percentages of bone-marrow B cells and absolute B cell counts in blood. Both variables are log-transformed, base-2

Absolute lymphocyte counts and absolute B cell counts were strongly associated with morphological bone-marrow involvement, and also with other markers for high tumor burden such as engagement of > 4 nodal areas (Table 2) and Ann Arbor stage as an ordinal variable (*P* < 0.0005 for both counts). Absolute lymphocyte counts and, particularly, absolute B cell counts correlated strongly with the relative B cell levels in bone marrow (*P* = 0.0003; *P* < 0.0005; Fig. 1). Higher absolute monocyte counts were associated with elevated LDH (Table 2). Absolute monocyte counts also co-varied positively with absolute lymphocyte counts (*P* < 0.0001), but not with absolute B cell counts or absolute T cell counts (*P* = 0.07; *P* = 0.56). Absolute B and T cell counts were also positively correlated (*P* = 0.0005).

**Table 2:** Associations between lymphocytes and monocytes and clinical variables

Variable	ALC		ABC		AMC	
	*P*	OR (95% CI)	*P*	OR (95% CI)	*P*	OR (95% CI)
Ann Arbor stage III–IV	0.089	1.78 (0.92–3.44)	0.175		0.67	
Involved nodal areas > 4	0.011	1.38 (1.08–1.76)	0.035	3.12 (1.08–8.99)	0.053	2.65 (0.99–7.15)
Hemoglobin ≤12 g/dL	0.89		0.60		0.73	
LDH > UNL	0.17		0.22		0.017	2.90 (1.21–7.00)
Bone marrow involvement	0.0001	2.11 (1.44–3.10)	0.023	54.9 (1.73–1739.3)	0.12	
Bulky disease	0.36		0.67		0.253	
B symptoms	0.68		0.60		1.00	
WHO grade 1	0.60		0.405			0.70

*ALC*, absolute lymphocyte count; *OR*, odds ratio; *CI*, confidence interval; *ABC*, absolute B cell count; *AMC*, absolute monocyte count; *LDH*, lactate dehydrogenase; *UNL*, upper normal limit; *WHO*, World Health Organization

### Lymphocytes and TNT

**Fig. 2:**
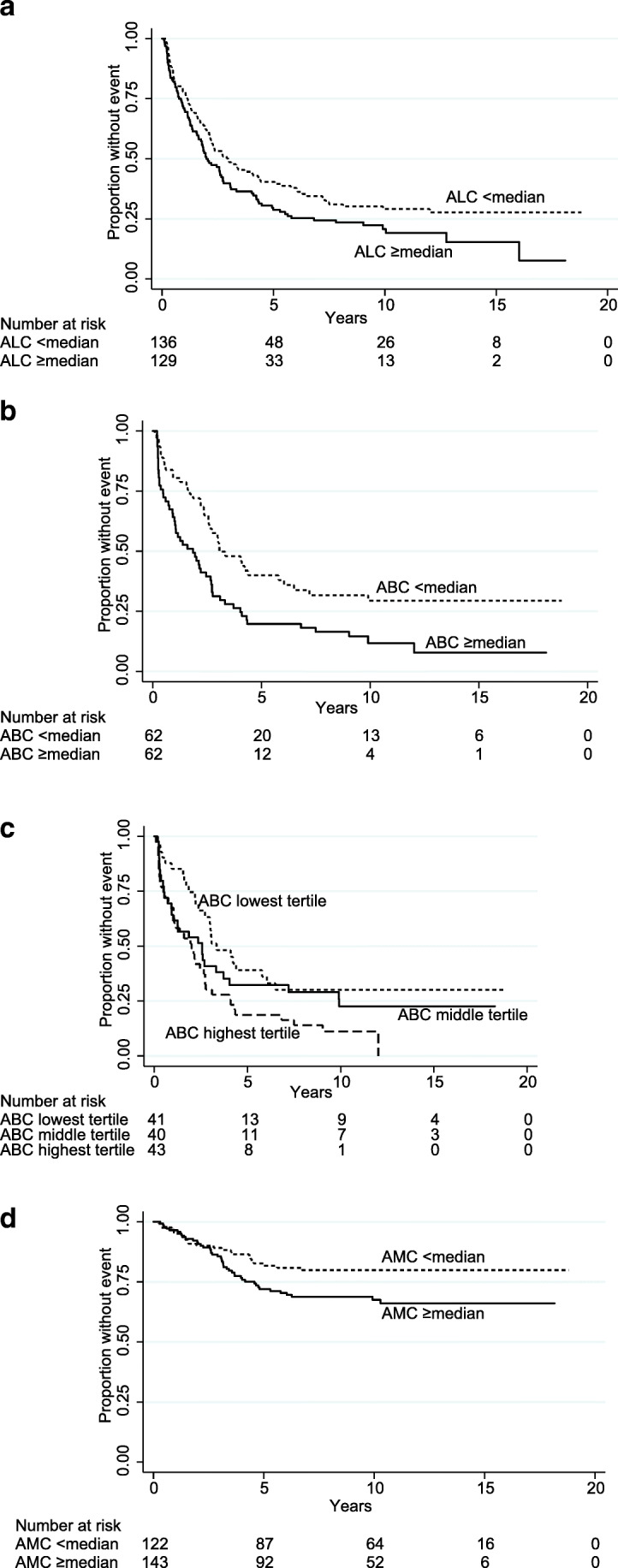
Outcome by lymphocytes and monocytes. Kaplan-Meier curves of time to next treatment or death by **a** absolute lymphocyte counts (ALC) divided by the median, **b** absolute B cell counts (ABC) divided by the median, **c** ABC divided into tertiles, and of **d** overall survival by absolute monocyte counts (AMC) divided by the median

Absolute lymphocyte counts ≥ median (1.3 × 10^9^/L) were associated with shorter TNT, median 2.0 versus 3.0 years (*P* = 0.041; Table 3, Fig. 2a); tertiles also predicted TNT (*P* = 0.008). The negative impact of absolute lymphocyte counts was due to B but not T or NK cells, because patients with absolute B cell counts ≥ median (0.09 × 10^9^/L) showed shorter TNT, median 1.9 versus 3.1 years (Table 3, Fig. 2b). Absolute T cell subset or NK cell counts did not predict outcome (Table 3). Relative T cell subset levels which in this material were previously shown be prognostic [5] were now outcompeted by the absolute B cell counts in multivariable analysis. The 19 patients with overt (> 4 × 10^9^/L) lymphocytosis (median [range] 7.3 [4.1–124.0] × 10^9^/L), showed inferior TNT (Table 1). The lymphocytosis was in all available cases (*n* = 14) due to elevated B cells. Also when these 19 patients were excluded from the analysis, absolute B cell counts remained significant for TNT (*P* = 0.011). Bone-marrow involvement or the relative levels of B cells in bone marrow did not correlate with TNT (*P* = 0.46; *P* = 0.39).

**Table 3:** Distributions of lymphocytes and monocytes and their relations to TNT and OS

Variable	*N*	Median	Range	p33	p66	TNT		OS	
						*P*	HR (95% CI)	*P*	HR (95% CI)
ALC × 10^9^/L	265	1.3	0.31–124.0	1.1	1.6	0.041	1.34 (1.01–1.79)	0.29	1.29 (0.80–2.09)
ABC × 10^9^/L	124	0.09	0.00–121.5	0.05	0.18	0.003	1.89 (1.25–2.86)	0.16	1.65 (0.82–3.31)
AMC × 10^9^/L	265	0.50	0.07–3.7	0.40	0.60	0.15	1.23 (0.93–1.64)	0.036	1.72 (1.04–2.84)
CD3+ × 10^9^/L	121	0.75	0.03–5.3	0.50	0.98	0.93	1.02 (0.68–1.54)	0.21	1.57 (0.77–3.18)
CD4+ × 10^9^/L	115	0.45	0.02–2.7	0.30	0.57	0.81	0.95 (0.62–1.45)	0.31	1.46 (0.71–3.00)
CD8+ × 10^9^/L	115	0.33	0.02–2.8	0.24	0.45	0.22	1.30 (0.85–1.99)	0.08	1.96 (0.93–4.13)
CD56+ × 10^9^/L	97	0.24	0.02–4.1	0.17	0.32	0.43	1.21 (0.76–1.93)	0.21	1.65 (0.75–3.64)

*TNT*, time to next treatment or death; *OS*, overall survival; *HR*, hazard ratio; *CI*, confidence interval; *ALC*, absolute lymphocyte count; *ABC*, absolute B cell count; *AMC*, absolute monocyte count

Analysis of tertiles (cut-offs in Table 3) of absolute B cell counts showed that the adverse effect on TNT increased incrementally (*P* = 0.005; Fig. 2c). Patients with absolute B cell counts in the lowest tertile had median TNT of 3.3 years while those in the middle and the highest tertiles had 2.6 and 2.0 years respectively. Relative B cell levels in blood ≥ median (7%) were also associated with inferior TNT (*P* = 0.013), but multivariable analysis showed that relative B cell levels were irrelevant when competing with absolute B cell counts. Absolute monocyte counts divided by the median (0.5 × 10^9^/L) and in tertiles were not significant for TNT (Table 3).

**Fig. 3:**
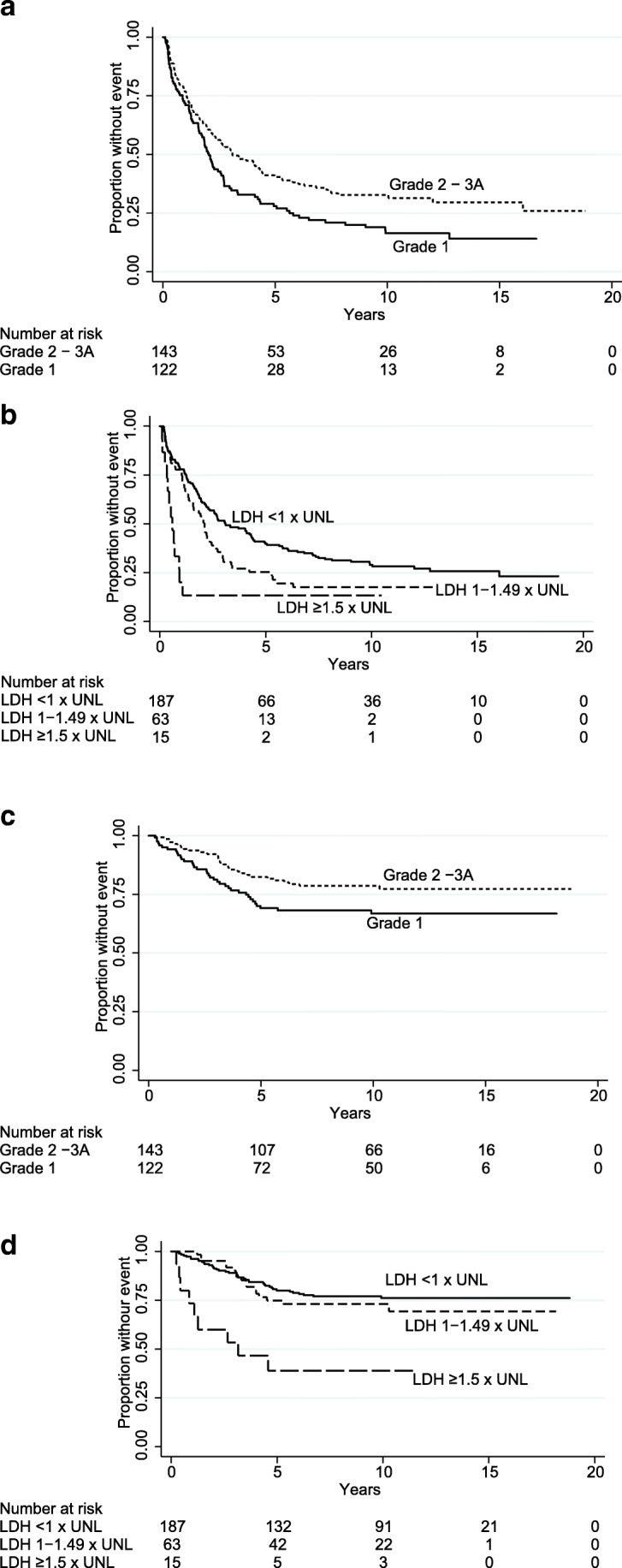
Outcome by follicular lymphoma grades and lactate dehydrogenase. Kaplan-Meier curves of time to next treatment or death by **a** follicular lymphoma grades and **b** lactate dehydrogenase (LDH) and of overall survival by **c** follicular lymphoma grades and **d** LDH

WHO FL grade 1 was associated with shorter TNT (Table 1; Fig. 3); patients with WHO grade 1 showed a median TNT of 2.0 years compared with 3.1 years in those with WHO grade 2–3A. WHO FL grade 1 also correlated with inferior OS (*P* = 0.033; Table 1; Fig. 3). The prognostic impact of high LDH was incremental, and particularly patients with LDH ≥ 1.50 × UNL showed inferior TNT (*P* = 0.0001; Fig. 3). The median TNT for this group was only 0.6 years while patients with LDH 1–1.49 × UNL and those with LDH < UNL had median TNT of 2.1 (*P* = 0.023) and 2.7 years respectively. Also, OS was significantly shortened in the group with LDH ≥ 1.50 × UNL (*P* = 0.0001; Fig. 3). Five-year OS was 39% in those with LDH ≥1.50 × UNL, 75% in the group with LDH 1–1.49 × UNL, and 80% in patients with LDH < UNL. LDH ≥ 1.50 × UNL was also significant in multivariable analysis for OS (*P* = 0.004). Elevated LDH outcompeted the FLIPI in the multivariable analysis for TNT.

In multivariable analysis with respect to TNT, absolute B cell counts ≥ median, WHO FL grade 1, and LDH > UNL were independent (Table 4). Absolute B cell counts predicted TNT both in patients treated with and without IFN (HR 1.6 and 2.1 respectively), although the reduced numbers decreased statistical significance (*P* = 0.13; *P* = 0.007, respectively).

**Table 4:** Multivariable analysis with respect to TNT

Independent variable	HR	95% CI	*P* value
ABC above the median (0.09 × 10^9^/L)	1.73	1.14–2.63	0.010
WHO grade 1	1.66	1.10–2.52	0.017
LDH > UNL	2.19	1.39–3.44	0.001

Competing, insignificant variables: B symptoms and WHO performance status

*TNT*, time to next treatment; *HR*, hazard ratio; *CI*, confidence interval; *ABC*, absolute B cell count; *WHO*, World Health Organization; *LDH*, lactate dehydrogenase; *UNL*, upper normal limit

### OS analysis

Absolute lymphocyte or B cell counts had no impact on OS (Table 3). Absolute monocyte counts ≥ median were associated with inferior OS (*P* = 0.036; Table 3; Fig. 2d) in univariate but not in multivariable analysis (data not shown). Absolute monocyte counts in tertiles were not significant (*P* = 0.29). We also tested the different cut-off values for absolute monocyte counts used by others: 0.57 × 10^9^/L [7], 0.34 × 10^9^/L [24], and 0.63 × 10^9^/L [25] but found none of them prognostic for TNT or OS in our cohort (data not shown).

## Discussion

In this analysis of FL patients, uniformly treated with rituximab without chemotherapy in two NLG trials and with long follow-up times (median 10.6 years), we show that higher pre-treatment absolute lymphocyte counts correlate with shorter TNT and that this is wholly attributable to the absolute numbers of B cells. In multivariable analysis for TNT, absolute B cell counts were independent of other prognostic variables. Furthermore, absolute B cell counts were prognostic also when excluding patients with lymphocytosis.

Absolute B cell counts correlated strongly with B cell levels in the bone marrow (Fig. 1) and also with other markers of high tumor burden (stage, number of nodal stations, bone-marrow involvement). However, the markers of high tumor burden did not correlate with outcome and absolute B cell counts remained significant in multivariable analysis. High absolute B cell counts probably reflect circulating lymphoma cells, but we did not have information on clonality. PET/CT was not conducted in these trials, which would have been a more ideal method of assessing burden of disease.

In previous reports, low absolute lymphocyte counts before rituximab monotherapy have been associated with outcome in FL [12–14]. In patients receiving rituximab monotherapy, Behl et al. reported that pre-treatment absolute lymphocyte counts < 0.89 × 10^9^/L (median of the study population) were associated with inferior treatment responses and shorter remissions [13], whereas Ghielmini et al. showed that pre-treatment absolute lymphocyte counts < 0.8 × 10^9^/L predicted superior responses to rituximab induction [14]. In these studies, however, most patients had received previous chemotherapy, and in the study of Ghielmini et al. patients with mantle cell lymphoma were also included (~ 30%). The cut-off values used by Behl et al. and Ghielmini et al. were not prognostic in our trials (data not shown).

Circulating lymphoma cells in FL are not heavily studied, and consistent criteria for defining peripheral blood involvement in FL are lacking [26]. Circulating lymphoma cells identified by morphology and/or flow cytometry and absolute neoplastic lymphocytosis (circulating lymphoma cells > 4 × 10^9^/L) have been investigated [26–28]. Studies in the rituximab era have shown that peripheral blood involvement has a negative impact on progression-free survival (PFS), time to progression, and TNT but no significant effect on OS [27–30]. In the study of Kodaira et al., leukemic presentation was defined by cytological identification of an abnormal lymphoid population in peripheral blood [29]. Maeshima et al. required ≥ 1% typical small-cleaved cells in the blood smear or positive flow-cytometry results and 11% consecutive FL patients thus showed peripheral blood involvement, with significantly shorter progression-free survival after rituximab-containing therapy, while OS did not differ between the groups [27]. The levels of circulating lymphoma cells were highly variable (range, 2–92%); notably, also low levels of circulating lymphoma cells predicted outcome, agreeing with our results [27]. It should be noted that we found absolute B cell counts to be prognostic also in patients with normal lymphocyte counts (< 4 × 10^9^/L), suggesting that small amounts of circulating malignant cells could be detrimental when treating patients with rituximab, and that they would not be identified via standard blood chemistry. Sarkozy et al. identified 37 patients with leukemic disease (7.4% of all), detected by blood smear analysis and confirmed by flow cytometry, and showed inferior outcome compared with matched patients without peripheral blood involvement. These results were also validated in the PRIMA cohort [28], in which the presence of circulation lymphoma cells was assessed prospectively by the local laboratories, but the technical modalities used for detection were not pre-specified. In a subsequent study of the PRIMA cohort, Sarkozy et al. also showed that the negative impact of peripheral blood involvement on PFS and TNT was obviated with rituximab maintenance [30]. Rituximab dosing and the length of maintenance therapy might be of particular importance in patients with peripheral blood involvement as high level of circulating B cells at baseline and bone-marrow involvement have, in some studies, been associated with lower rituximab concentrations and inferior treatment outcomes in FL [31, 32].

In our cohort, high absolute monocyte counts were associated with shorter OS in univariate but not multivariable analysis. Furthermore, absolute monocyte counts were not prognostic when divided into tertiles. There was no significant association between absolute monocyte counts ≥ median and TNT, why we also tested the different cut-off values used by others: 0.57 × 10^9^/L [7], 0.34 × 10^9^/L [24], and 0.63 × 10^9^/L [25] but found none of them prognostic for neither TNT nor OS. Wilcox et al. first demonstrated that higher absolute monocyte count at diagnosis was an independent predictor for poor OS in a heterogeneous cohort of FL patients, of whom 25% received rituximab containing therapy and 45% were initially observed [7]. In a subgroup analysis of patients with immediate treatment indication, no association between higher absolute monocyte count and OS was found [7]. The results from subsequent studies on the prognostic significance of absolute monocyte counts (at diagnosis) in FL patients treated with immunochemotherapy, are conflicting [24, 25]. Patient selection and treatment regimens may explain the contradictory results of these studies. We do not think that absolute monocyte counts at initiation of rituximab therapy are of independent prognostic value.

Press et al. have previously suggested a laboratory test-based prognostic model consisting of LDH and β_2_-microglobulin in patients treated with immunochemotherapy or chemotherapy followed by radioimmunotherapy, with an optimal cut-off point for LDH (and β_2_-microglobulin) of 150% of the UNL [33]. We investigated this cut point in our patient population, and the group with LDH ≥ 1.50 × UNL had very short TNT (median 0.6 years) and inferior OS which makes rituximab without chemotherapy an inappropriate choice of first-line therapy for this category. For this report we did not have β_2_-microglobulin levels, but based on our LDH results and those shown for β_2_-microglobulin in the PRIMA-PI [34], we plan to use LDH ≥ 1.50 × UNL and β_2_-microglobulin > 3 mg/L for stratification in future NLG trials.

The main strengths of the present analysis are the large sample size of FL patients uniformly treated with rituximab without chemotherapy, the long follow-up times, and additional information on lymphocyte subsets by the use of flow cytometry. We did not have flow cytometry profiles on all patients, which limits the assessment of the simultaneous impact of lymphocyte subsets and other factors on the outcome. Yet, neither outcome measures nor other prognostic variables differed significantly between those with available flow cytometry profiles and those with missing flow cytometry. Furthermore, markers for clonality or CD10 were not available, which makes it possible that these excess circulating B cells are non-malignant. However, the strong co-variations between absolute B cell counts and bone-marrow B cell levels (and morphological bone-marrow involvement) support our assumption that the peripheral B cells are mostly malignant. Since the exclusion of patients with lymphocytosis did not change the main results, even small numbers of circulating B cells appear deleterious for outcome.

In conclusion, we found that higher pre-treatment absolute B cell counts in blood were independently associated with shorter TNT in FL patients treated with rituximab without chemotherapy. Thus, treatment with rituximab in conventional doses appears insufficient for FL patients with higher absolute B cell counts. The negative prognostic impact of higher absolute B cell counts, also in small amounts, likely reflects the impact of circulating lymphoma cells. Studies with more detailed flow cytometry markers and on patients receiving chemoimmunotherapy are needed to further explore these cells and their prognostic/predictive properties in FL.

## Data Availability

The datasets used during the current study are available from the corresponding author on reasonable request.

## List of abbreviations

B2M: β_2_-microglobulin
FL: Follicular lymphoma
FLIPI: Follicular Lymphoma International Prognostic Index
IFN: Interferon-α2a
LDH: Lactate dehydrogenase
NLG: Nordic Lymphoma Group
OS: Overall survival
PFS: Progression-free survival
UNL: Upper normal limit
TNT: Time to next treatment or death
WHO: World Health Organization
